# Transient changes in body weight and behavior during the placentation period in non-human primates and rodents

**DOI:** 10.1038/s41598-026-41314-8

**Published:** 2026-03-25

**Authors:** Saori Yano-Nashimoto, Kazutaka Shinozuka, Takuma Kurachi, Katsura Kagawa, Kentaro Q. Sakamoto, Yuko Shigeno, Kimie Niimi, Kumi O. Kuroda, Soichiro Yamaguchi

**Affiliations:** 1 Laboratory of Physiology, Department of Basic Veterinary Sciences, Faculty of Veterinary Medicine, Hokkaido University, Kita 18, Nishi 9, Kita-ku, Sapporo, Hokkaido 060-0818 Japan; 2 Laboratory for Affiliative Social Behavior, RIKEN Center for Brain Science, Wako, Saitama 351-0198 Japan; 3 Department of Agriculture, Tokyo University of Agriculture and Technology, Fuchu, Tokyo, 183-8509 Japan; 4 Atmosphere and Ocean Research Institute, The University of Tokyo, Kashiwa, Chiba 277-8564 Japan; 5 Support Unit for Animal Resources Development, Research Resources Division, RIKEN Center for Brain Science, Wako, Saitama 351-0198 Japan; 6 Kuroda Laboratory, School of Life Science and Technology, Institute of Science Tokyo, Yokohama, Kanagawa 226-8503 Japan

**Keywords:** Morning sickness, Nausea and vomiting of pregnancy, Mouse, Common marmoset, Ecology, Ecology, Neuroscience, Physiology, Zoology

## Abstract

During early pregnancy, many women experience physical changes, including nausea and vomiting of pregnancy (NVP), which negatively impact their quality of life. However, the absence of model animals has limited our understanding of such pregnancy-associated physiological changes. Here, we examined pregnancy-associated metabolic and behavioral changes in common marmosets and mice. Marmosets exhibited a transient weight decrease during the period of placental development in approximately 22% of pregnancies. Some marmosets repeatedly showed transient weight loss across multiple pregnancies, suggesting individual variations in the likelihood of pregnancy-associated weight loss. Although mice did not show apparent alteration in body weight, they exhibited a slowdown in food intake and alterations in locomotor activity during the corresponding phase. The observed transient changes in pregnant marmosets and mice may provide a basis for generating hypotheses regarding physiological changes associated with placentation.

## Introduction

During early pregnancy, many women experience physical changes, including nausea, vomiting, anorexia, weight decrease, malaise, and alterations in taste and olfaction. These symptoms are commonly referred to as “morning sickness,” among which nausea and vomiting are clinically recognized as nausea and vomiting of pregnancy (NVP). Pregnancy-associated symptoms are typically most severe during the first trimester, when placental development is most active. NVP affects approximately 70–80% of pregnant women and impairs their quality of life^1–3^. In severe cases, NVP has been associated with adverse pregnancy outcomes, including preterm birth, low birthweight, and fetal growth restriction, and some studies have reported potential longer-term impacts on offspring, such as neurodevelopmental delay^4^. However, the underlying mechanisms remain poorly understood, and thus no definitive treatment currently exists^2,5^.

One major obstacle to elucidating the mechanisms of NVP is the lack of appropriate non-human animal models. Several mammalian species have been reported to show pregnancy-associated changes reminiscent of those observed in pregnant women^6,7^. For example, dogs may show reduced food intake and occasional vomiting during early pregnancy^6^. Pregnant rhesus monkeys have been observed to reject food more frequently^7^. However, because these observations have not been followed by systematic scientific investigation, it is unclear whether these alterations progress to more severe symptoms such as weight loss, and whether the timing corresponds to specific stages of human gestation. Thus, these phenomena have not been directly established as equivalent to pregnancy-associated symptoms in humans.

Here, we systematically examined pregnancy-associated metabolic and behavioral changes in common marmosets (*Callithrix jacchus*) and mice (*Mus musculus*), two species widely used in biological research. As direct evaluation of nausea and vomiting is challenging in mice and in marmosets housed in family groups, we instead monitored physiological and behavioral indices such as body weight, food intake, and locomotor activity. Using this approach, we found that some individuals in marmosets exhibited a transient decrease in body weight during the period of placental development. Furthermore, in mice, we observed a transient slowdown in food intake and alterations in locomotor activity during the corresponding gestational phase. Together, these observations reveal previously undercharacterized pregnancy-associated changes in non-human mammals and provide a basis for generating hypotheses about physiological alterations during placentation.

## Results

### Transient weight loss during mid-pregnancy was revealed by cluster analysis in pregnant marmosets

**Fig. 1:**
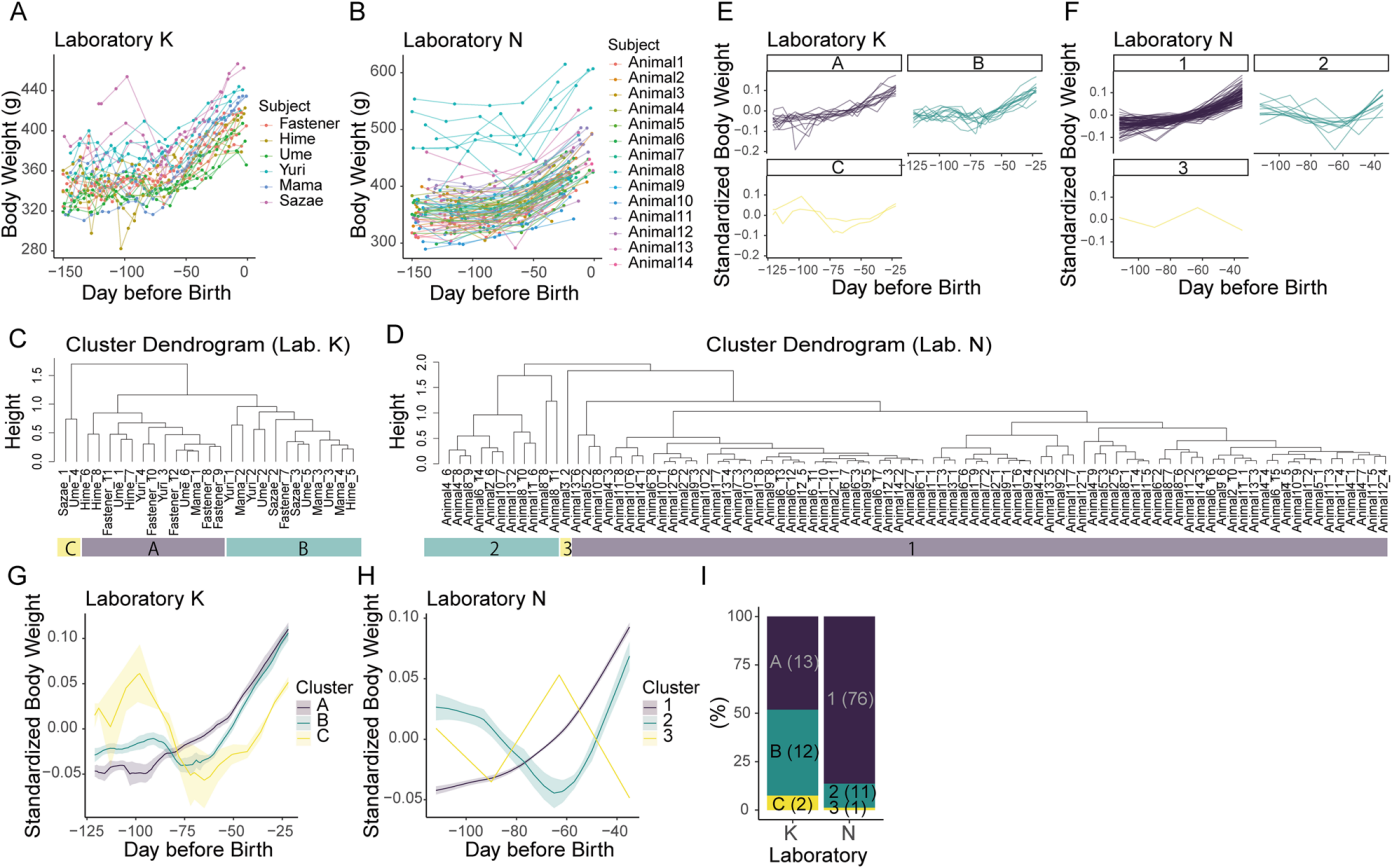
Clustering of body weight changes in pregnant marmosets. (**A**, **B**) Body weight changes in pregnant marmosets from two laboratories (Laboratory K and N). The x-axis shows days before birth (Day 0). (**C**, **D**) Cluster dendrograms based on pairwise correlations of body weight trajectories during pregnancy. Each leaf represents a pregnancy. The height of each node corresponds to the dissimilarity (1 – correlation coefficient) at which clusters are joined. Cluster labels (A–C or 1–3) are indicated below the leaves. (**E**–**H**) Body weight changes during pregnancy, shown separately for each cluster. For each pregnancy, body weight was standardized by its mean value. In panels E and F, each line represents a pregnancy. Cluster labels (A–C or 1–3) are indicated above each small panel. In panels G and H, the mean ± standard error is shown in each cluster. (**I**) The percentage of each cluster in each laboratory. The numbers within parentheses are the numbers of pregnancies included in the cluster. The proportion of pregnancies assigned to each cluster is shown for descriptive purposes only. Due to differences in body weight measurement frequency between laboratories, quantitative comparisons of cluster proportions across laboratories were not performed.

We conducted a cluster analysis on the body weight changes in pregnant marmosets from two different laboratories: Laboratory K (6 mothers, 27 pregnancies) and Laboratory N (14 mothers, 88 pregnancies) (Fig. 1A, B). The data from these laboratories were analyzed separately because the frequency of weight measurements differed. Hierarchical clustering was performed based on pairwise correlation dissimilarities. The data were grouped into three clusters by cutting the dendrogram (Fig. 1C–H). In Laboratory K, 13 pregnancies were classified as cluster A, which showed a consistent increase in body weight (Fig. 1C, E, G, I). Twelve and two pregnancies were classified as cluster B and C, respectively, both of which exhibited a transient decrease during mid-pregnancy, approximately 95–65 days before delivery. Similarly, in Laboratory N, the largest cluster (Cluster 1) showed a consistent increase, while the second cluster (Cluster 2) showed a transient decrease during mid-pregnancy (Fig. 1D, F, H, I). Notably, the timing of weight decrease was strikingly similar across Cluster B, C, and 2 (Fig. 1G, H), and this period corresponds to the phase of placental development in marmosets^8,9^.

**Fig. 2:**
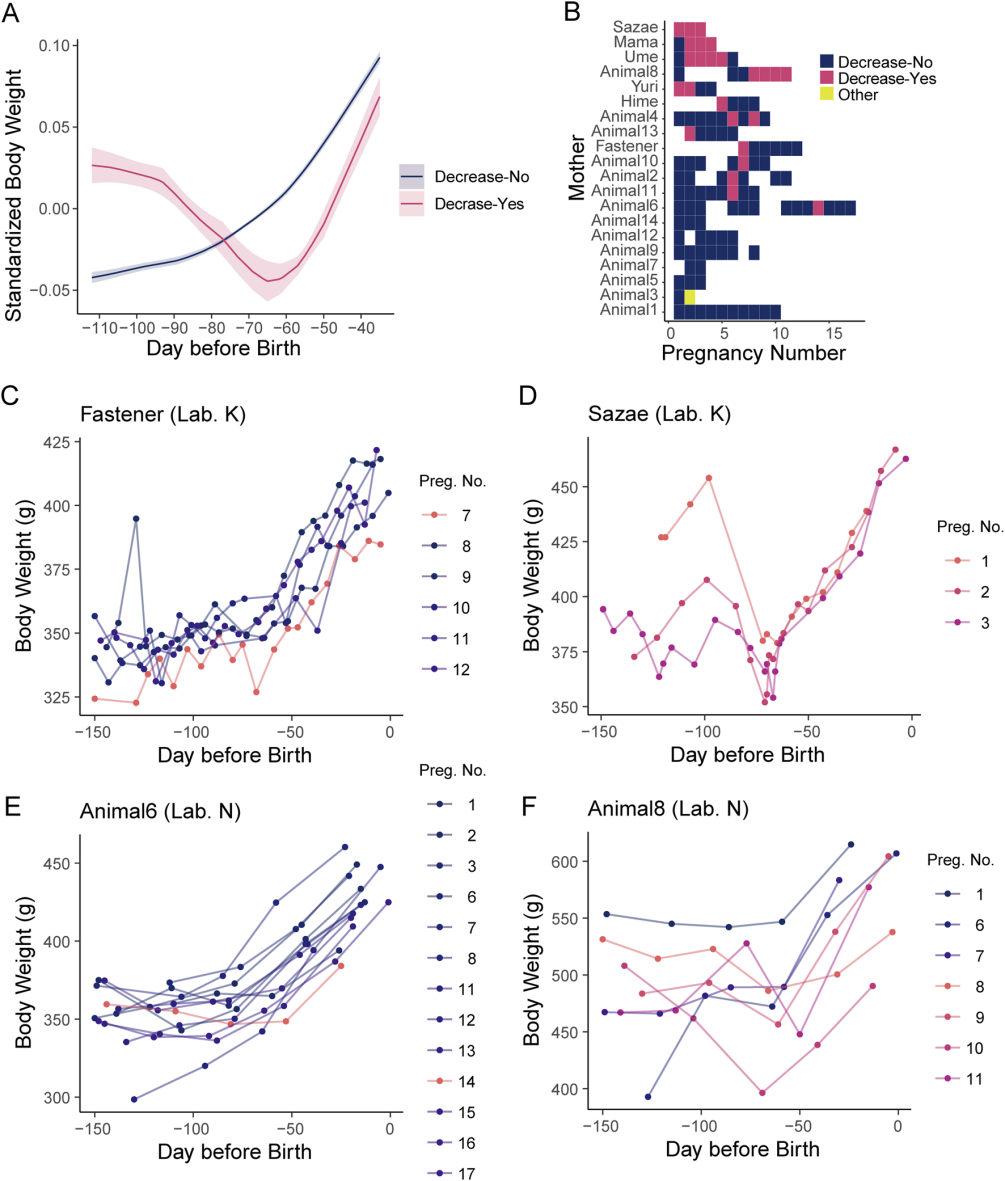
A subset of marmoset mothers repeatedly exhibited transient decreases in body weight during mid-pregnancy. (**A**) Body weight changes during pregnancy, shown separately for two superclusters: “Decrease-No”, and “Decrease-Yes”. “Decrease-No” includes Cluster A and Cluster 1; “Decrease-Yes” includes Cluster B, C, and 2. Cluster 3 is classified as “Other” and not shown in this panel. Body weight was standardized by its mean value. The mean ± standard error is shown in each cluster. (**B**) Tile plot showing cluster classification for each mother and pregnancy. Colors indicate cluster membership. (**C**–**F**) Body weight changes during pregnancy in representative mothers. The colors indicate cluster classification: red for "Decrease-Yes" and blue for "Decrease-No." Panels C and E show mothers who were less likely to experience weight loss during pregnancy. Panels D and F show mothers who frequently lost weight during pregnancy. Mothers in panels C and D are from Laboratory K, and those in panels E and F are from Laboratory N.

Given the similarity in cluster characteristics across the two laboratories, we combined the data and re-classified them into two superclusters: Decrease-No and Decrease-Yes. Decrease-No includes Cluster A and 1, while Decrease-Yes includes Cluster B, C, and 2 (Fig. 2A, B). Cluster 3, which included only one pregnancy, was classified as “Other”. From 95 to 65 days before birth, body weight in the Decrease-No group increased by 3.4 ± 0.3% relative to the average pregnancy weight. In contrast, the Decrease-Yes group showed a decrease of 6.3 ± 1.5% during the same period, followed by a subsequent increase (Fig. 2A). There was individual variation in how often transient weight loss occurred during mid-pregnancy (Fig. 2B–F). Although the proportion of Decrease-Yes pregnancies differed between Laboratory K and N (Figs. 1I, 2B), some of the mothers showed this pattern repeatedly (Fig. 2D, F), whereas others rarely did (Fig. 2C, E).

### Mice showed transient alterations in food intake and locomotion during mid-pregnancy

**Fig. 3:**
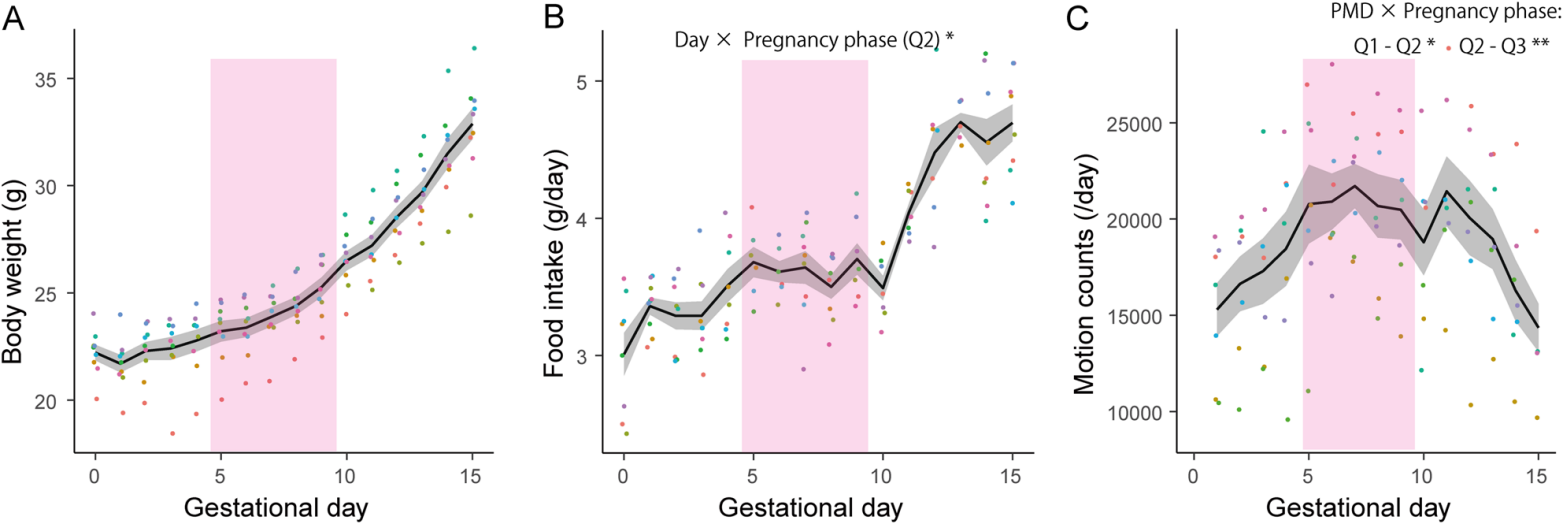
Mice exhibited transient alterations in food intake and locomotion during mid-pregnancy. (**A**) Body weight changes during pregnancy. Body weight increased throughout pregnancy. (**B**) Changes in food intake during pregnancy. Although food intake increased throughout pregnancy, the rate of increase slowed during the second quarter of pregnancy (Q2). *LMM, *p* < 0.05. (**C**) Changes in locomotor activity during pregnancy. Locomotor activity increased according to the progress of gestation during the first quarter of pregnancy, plateaued during Q2, and then declined during the third quarter. LMM, **p < 0.01, *p < 0.05. The mean ± standard error is shown as a black line with a gray shaded area. The color of each dot represents an individual mouse. Pink shading indicates Q2 period (gestational day 5–9).

To examine whether similar alterations occur in mice, we measured the body weight, food intake, and locomotor activity during pregnancy. Because pregnancy-associated changes in humans, including NVP, as well as transient weight decrease in marmosets are observed during the phase of placental development, we compared physical and behavioral parameters during the second quarter of pregnancy (Q2), when placentation progresses^10^, with those in other gestational phases. Body weight increased throughout pregnancy and showed no apparent alteration during Q2 (Fig. 3A). Meanwhile, although the food intake increased throughout pregnancy, the rate of increase slowed during Q2 (Fig. 3B). Locomotor activity increased according to the progress of gestation during the first quarter of pregnancy, plateaued during Q2, and then declined during the third quarter, when weight gain became more pronounced (Fig. 3A, C). In other words, the slowdown of the increase in food intake and locomotor activity was observed during Q2.

## Discussion

In the present study, we identified pregnancy-associated changes in two different mammals. In marmosets, a transient decrease in body weight occurred in a subset of pregnancies during mid-pregnancy. In mice, although no body weight decrease was detected, the increase in food intake and locomotor activity slowed during the second quarter of pregnancy. Importantly, both periods when these alterations occurred in the two species were consistent with placental development^8–10^. In addition, although different parameters were affected in the species, the reductions in body weight, food intake, and locomotion are commonly discussed in the context of decreased physical condition ^11–13^. Similar pregnancy-associated changes in non-human mammals have been mentioned sporadically and largely in an anecdotal manner, often based on isolated observations by zookeepers or animal caretakers, occasionally prompting speculation that physiological states resembling those experienced by pregnant women may also occur in other species. However, such observations have not been systematically examined, and scientific investigations addressing these phenomena remain limited^6,7^. The present study provides a systematic, quantitative description of pregnancy-associated changes based on multiple individuals and extends beyond a single species by reporting comparable alterations in two mammalian species. Based on these observations, we propose the hypothesis that pregnancy-associated physiological changes, which may resemble aspects of early pregnancy conditions in humans, could be widespread among non-human mammals.

Physiological changes during early pregnancy in humans are diverse, including nausea, vomiting, anorexia, weight decrease, malaise, and alterations in taste and olfaction. The most characteristic symptom during early pregnancy is vomiting^14,15^, which cannot be assessed in the present study because mice do not vomit^11^ and because our marmoset subjects were kept in family groups. Since marmosets sometimes vomit, it may be of interest to investigate their occurrence in pregnant marmosets in future research. Alterations in taste and odor in pregnant animals were reported in several studies^16–18^, but the timing does not fully correspond to the gestational stage of human NVP. Thus, it should be carefully considered whether these alterations were related to phenomena observed in humans. In addition, given the diverse symptoms in pregnant women, it is possible that different symptoms are driven by distinct mechanisms^19^, underscoring the need for symptom-specific investigations.

In marmosets, transient weight decreases were observed only in approximately 22% of pregnancies, rather than universally. In addition, some marmoset females repeatedly exhibited transient weight loss across multiple pregnancies, suggesting the possibility of individual variations in the likelihood and severity of pregnancy-associated changes. In mice, such variation was not observed, possibly due to their uniform genetic background. While previous studies have examined changes in food intake and activity levels during pregnancy in mice^20,21^, alterations in food intake and locomotion during the second quarter of pregnancy have received little attention. This may be because the changes are relatively mild and therefore difficult to detect. In addition, differences in methods for assessing activity, such as running wheel activity versus spontaneous movement in a home cage, may influence outcomes^21^. Strain differences might also contribute to the variability and make it more difficult to identify these subtle alterations.

In the present study, pregnancy-associated changes were observed in different species at a similar gestational period corresponding to placental development. If the alterations observed in marmosets and mice reflect physiological states that are analogous to those experienced during early pregnancy in humans, focusing on the placentation period may provide a useful starting point for future mechanistic studies. However, species differences must be considered carefully^22^. Hormonal dynamics during pregnancy differ across species: marmosets produce mCG instead of hCG^23^, and mice lack chorionic gonadotropin (CG) but secrete placental prolactin-like hormones (PL-I, PL-II)^24^. The timing of estrogen and progesterone rise also varies^22,25^. Moreover, while all three species have hemochorial placentas, the structure of the maternal–fetal barrier differs, with humans having the thinnest, mice the thickest, and marmosets showing an intermediate structure^8,22^. For example, one proposed cause of NVP, Growth Differentiation Factor 15 (GDF15), is released from the fetus^26,27^, suggesting that differences in placental structure and maternal–fetal exchange may need to be considered when interpreting pregnancy-associated physiological changes across species. Interestingly, the increase in GDF15 levels during pregnancy is lower in mice than in humans and non-human primates^28,29^. However, the relationship between these molecules, including GDF15, and the pregnancy-associated changes observed in this study was not directly examined. Further studies will be required to elucidate the biological basis of pregnancy-associated symptoms.

## Methods

### Marmosets

All experiments using common marmosets were approved by the Animal Experiment Judging Committee of RIKEN (equivalent of Institutional Animal Care and Use Committee, IACUC, approval numbers H28-2-210, H30-2-206, W2020-2-027, H25-2-212, H27-2-212, H29-2-211, W2019-2-011, and W2021-2-033) and were conducted in accordance with the 2011 guideline from the National Research Council of the National Academies. Common marmosets were reared at the RIKEN Center for Brain Science in accordance with the institutional guidelines and under veterinarians’ supervision. This study is reported in accordance with the ARRIVE guidelines.

We used 20 adult female marmosets from two different laboratories: Laboratory K (6 mothers, 27 pregnancies) and Laboratory N (14 mothers, 88 pregnancies). Each female was housed in a family cage with her husband and offspring (0–6 individuals per cage). Body weight was measured regularly (Laboratory K: once a week, Laboratory N: once a month) and additionally as needed for health assessments. Water and food were supplied ad libitum. The monkeys’ food was replenished at approximately 11:30, and supplementary foods, such as a piece of sponge cake, dried fruits, and lactobacillus preparation, were given in the afternoon without a fixed schedule. The photoperiod of the colony room was 12L:12D (light period: 8:00–20:00, dark period: 20:00–8:00). Measurements were conducted between 8:00 and 17:00.

### Clustering analysis

We collected data on the body weight of pregnant females between 150 and 1 day before delivery. Pregnancies were excluded if they had insufficient measurements (i.e., the first measurement was later than 100 days before delivery) or if they resulted in miscarriage or stillbirth. A total of 115 pregnancies (Laboratory K: 27, Laboratory N: 88) were included in the clustering analysis.

Cluster analysis was conducted using R (version 4.4.3)^30^. First, missing body weight values were estimated by linearly connecting the data points to allow analysis of continuous weight patterns. The data from two laboratories were analyzed separately because the frequency of weight measurements differed. To ensure complete data for analysis, we used only the time points for which body weight measurements were available for all pregnancies (Laboratory K: between 121 and 22 days before delivery, Laboratory N: between 112 and 35 days before delivery). The body weight trajectories for each pregnancy were standardized by dividing each data point by the mean weight of that pregnancy. To quantify the pairwise similarity between trajectories, we calculated correlation-based dissimilarities using the *diss* function from the *TSclust* package, with the method set to “COR”^31^. Hierarchical clustering was then performed using the *hclust* function with the default setting. Dendrograms were visually inspected, and clusters were defined by cutting the tree at a height that yielded three major groups. These clusters were named based on cluster size: Cluster A, B, and C in Laboratory K, and as Cluster 1, 2, and 3 in Laboratory N. These clusters were then used to characterize common patterns of weight change during pregnancy.

Reclassification into two superclusters was performed based on the similarity in the two laboratories as follows: Decrease-No and Decrease-Yes. Pregnancies in Decrease-No included Cluster A and Cluster 1, both of which showed continuous body weight increases throughout pregnancies. Decrease-Yes included Cluster B, C, and 2, which showed a transient body weight decrease during mid-pregnancy. Cluster 3, which included only one pregnancy, was classified as “Other”. Data were analyzed between 112 and 35 days before delivery, as all data were available.

### Mice

All experiments using mice were approved by the Institutional Animal Care and Use Committee of the Faculty of Veterinary Medicine, Hokkaido University (approval numbers 20-0066 and 23-0026). Animals were handled in accordance with the Guide for the Care and Use of Laboratory Animals, Faculty of Veterinary Medicine, Hokkaido University (approved by the Association for Assessment and Accreditation of Laboratory Animal Care International). This study is reported in accordance with the ARRIVE guidelines. Water and food were supplied ad libitum. The photoperiod was 12L:12D (light period: 7:00–19:00, dark period: 19:00–7:00).

We used a total of 16 adult C57BL/6JJcl female mice for the experiments. These mice were co-housed with male C57BL/6JJcl mice. The day on which a vaginal plug was observed was designated as gestational day 0 (Gd0), and then the female was singly housed. Pregnant phases were defined as follows. 1^st^ quarter of pregnancy (Q1): from Gd0 to Gd4, 2^nd^ quarter (Q2): from Gd5 to Gd9, 3^rd^ quarter (Q3): from Gd10 to Gd15.

### Measurement of body weight, food intake, and locomotor activity in mice

Nine pregnant mice were singly housed in standard home cages (170 × 280 × 120 mm). Four food pellets were placed on the perforated top of a box (80 × 120 × 250 mm) and kept in the home cage. At 11:00 each day, the food and box were replaced with new ones, and the body weight of each mouse was measured. Uneaten and spilled food was collected from the box, dried overnight, and weighed. Daily food intake was calculated based on the difference between the provided and recovered food.

Seven pregnant mice were singly housed in special cages (135 × 190 × 160 mm) with an activity sensor (AS-10, Melquest, Japan). Locomotor activity was continuously recorded, and total activity counts were calculated for each 24-h period.

### Statistical analysis

Statistical analysis was conducted using R (version 4.4.3)^30^. Cluster analysis in pregnant marmosets is performed as mentioned above. To examine changes in pregnant mice, we used a linear mixed-effects model (LMM) using the *lmer* function from the *lme4* and *lmerTest* packages^32^. Mouse identity was included as a random effect, with random slopes for the gestational day. For food intake, Q2 was included in the model as a binary variable because we hypothesized that Q2 (when placentation progresses) was a physiologically distinct period. In contrast, for locomotor activity, Q1-3 were included in the model as categorical fixed effects (with Q2 as the reference) to evaluate stage-wise changes across pregnancy. Fixed effects included gestational day, pregnancy phase (i.e., Q2 or Q1-3), and their interaction. Model selection was based on Akaike Information Criterion (AIC) using the *dredge* function from the *MuMIn* package^33^.

## Data Availability

The datasets used and/or analysed during the current study are available from the corresponding author on reasonable request.
